# Expression Patterns of Atlantic Sturgeon (*Acipenser oxyrinchus*) During Embryonic Development

**DOI:** 10.1534/g3.116.036699

**Published:** 2016-12-14

**Authors:** Elisavet Kaitetzidou, Arne Ludwig, Jörn Gessner, Elena Sarropoulou

**Affiliations:** * Institute for Marine Biology, Biotechnology and Aquaculture, Hellenic Centre for Marine Research, 71003 Heraklion, Crete, Greece; † School of Biology, Faculty of Science, Aristotle University of Thessaloniki, 54124, Greece; ‡ Evolutionary Genetics Research Group, Leibniz Institute for Zoo and Wildlife Research, 10315 Berlin, Germany; § Biology and Ecology of Fishes, Leibniz Institute for Freshwater Ecology and Inland Fisheries, 12587 Berlin, Germany

**Keywords:** RNA-seq, development, *Acipenser*, embryogenesis, hatching

## Abstract

During teleost ontogeny the larval and embryonic stages are key stages, since failure during this period of tissue differentiation may cause malformations, developmental delays, poor growth, and massive mortalities. Despite the rapid advances in sequencing technologies, the molecular backgrounds of the development of economically important but endangered fish species like the Atlantic sturgeon (*Acipenser oxyrinchus*) have not yet been thoroughly investigated. The current study examines the differential expression of transcripts involved in embryonic development of the Atlantic sturgeon. Addressing this goal, a reference transcriptome comprising eight stages was generated using an Illumina HiSequation 2500 platform. The constructed *de novo* assembly counted to 441,092 unfiltered and 179,564 filtered transcripts. Subsequently, the expression profile of four developmental stages ranging from early (gastrula) to late stages of prelarval development [2 d posthatching (dph)] were investigated applying an Illumina MiSeq platform. Differential expression analysis revealed distinct expression patterns among stages, especially between the two early and the two later stages. Transcripts upregulated at the two early stages were mainly enriched in transcripts linked to developmental processes, while transcripts expressed at the last two stages were mainly enriched in transcripts important to muscle contraction. Furthermore, important stage-specific expression has been detected for the hatching stage with transcripts enriched in molecule transport, and for the 2 dph stage with transcripts enriched in visual perception and lipid digestion. Our investigation represents a significant contribution to the understanding of Atlantic sturgeon embryonic development, and transcript characterization along with the differential expression results will significantly contribute to sturgeon research and aquaculture.

Sturgeons are commonly associated with black caviar, which derives from their roe and is considered as their main product. Besides the high economic value of caviar, sturgeons are also of significant commercial interest due to their boneless flesh. They belong to the family Acipenseridae, an early-diverging fish lineage that has long been thought to be a “living fossil” (Romer 1966; Gardiner 1984; Bemis *et al.* 1997; Rabosky *et al.* 2013). The Acipenseridae family comprises six genera and 27 species, *i.e.*, two monotypic paddlefish genera (*Psephurus* and *Polyodon*) and four sturgeon genera (*Huso*, *Scaphirhynchus*, *Pseudoscarphirhynchus*, and Acipenser. The genus *Acipenser* is the most frequent one, containing 17 species, while the genera *Huso*, *Scaphirhynchus*, and *Pseudoscarphirhynchus* consist of two, three, and three species, respectively (http://fishbase.org). Of all the Acipenserid species, 85% are being classified as endangered or threatened by extinction (International Union for Conservation of Nature 2015). As a result of the ancient separation from teleosts, which dates back over 250 MYA, the morphological stasis (Bemis *et al.* 1997), as well as the extremely slow rate of molecular evolution (Krieger and Fuerst 2002), sturgeons inhabit a leading position in evolutionary biology. From a phylogenetic point of view, of particular interest is the ploidy of sturgeons. Different levels of ploidy have been reported due to multiple and independent duplication events (Ludwig *et al.* 2001; Fontana *et al.* 2008) resulting in two main groups; one group of ∼120 and a second group of ∼240 chromosomes. Regarding the transcriptome level, several studies have been initiated in the Adriatic sturgeon (*Acipenser naccarii*) and the Amur Sturgeon (*A. schrenckii*) (Vidotto *et al.* 2013; Zhang *et al.* 2016), with the former one being organized via a public database (AnaccariiBase). Transcriptomic changes in reproductive tissues have been studied in the lake sturgeon, *A. fulvenscens* (Hale *et al.* 2009), while the transcriptome and expression profiles during embryonic and larval development in sturgeons has been addressed only recently in the Siberian sturgeon, *A. baerii* (Song *et al.* 2015). Larval and embryonic stages are the most sensitive period during ontogeny, since important developmental events take place early in development rendering the embryo susceptible to external physical or chemical stress. Any failure during the time of tissue differentiation may cause malformations, developmental delays, poor growth, and massive mortalities (Rice *et al.* 1987; Miller *et al.* 1988). Advanced understanding of molecular processes underlying embryonic and larval development may contribute to generate efficient and reliable molecular tools for improved larval rearing conditions, which in turn may support aquaculture and restocking efforts. It has further been shown that, during teleost development, the stage of incubation (embryonic development) (Falahatkar *et al.* 2014) as well as the transition from endogenous feeding to exogenous feeding with the associated morphological differentiation are critical (Hardy and Litvak 2004). The present study focuses on the transcriptome of early developmental stages of the Atlantic sturgeon (*A. oxyrinchus*) up until 2 d after hatching. *A. oxyrinchus* is distributed in North America from the Canadian rivers St. John and St. Lawrence in the North to the US rivers entering the Gulf of Mexico. They spend most of their life in marine water but spawn in freshwater (Smith 1985; Kynard and Horgan 2002; Stein *et al.* 2004). Historically, a population was founded in North Europe a few millennia ago that was extirpated due to river regulation, pollution, and overfishing in the 19th century (Ludwig *et al.* 2002, 2008). Today, *A. oxyrinchus* is considered “Near Threatened globally” (International Union for Conservation of Nature 2015). Since the 1990s, a number of restoration programs have been initiated out in several Baltic range countries. To investigate the transcriptome of early developmental stages of the Atlantic sturgeon, *de novo* transcriptome assembly was first performed comprising eight different developmental stages and the expression profiles of four representative developmental stages have subsequently been assessed. Sturgeons, belonging to the Acipenseriformes, have not experienced a third round of whole-genome duplication (3R WGD). Consequently, the expansion of molecular data in sturgeon is of importance to identify homologous genes that will contribute to phylogenetic analysis and, thus, will shed light onto the primordial function of the transcripts. In addition, our data will enhance the development of molecular tools to assess quality parameters supporting the growth of sturgeon aquaculture.

## Materials and Methods

### Ethical requirements

All procedures involving the handling and treatment of fish used during this study were approved by the Internal Committee for Ethics and Animal Welfare of the Leibniz-Institute for Zoo and Wildlife Research (IZW).

### Sampling

In total, eight developmental stages from sturgeon embryo (2 hpf) to 2 dph larvae, *i.e.*, before first cleavage (2 hpf), middle gastrula (22:50 hpf), gastrula complete/onset of neurulation (30.30 hpf), heart tube S-shaped, onset of heartbeat (59:20 hpf), hatch of advanced embryos (98:45 hpf), mass hatching (102:15 hpf), just after hatching (121:00 hpf), and 2 dph (154:20 hpf) were sampled by transferring the animals individually into ice-cold water for 30 sec before being transferred to sample vials with 5 ml RNAlater (QIAGEN, Hilden, Germany). Subsequently, the samples were stored at −80° for transcriptome analysis. Staging was performed according to Ginsburg and Dettlaff (1991). Each sampling point comprised a pool of seven individuals and was sampled in triplicate.

### Messenger RNA extraction

Messenger RNA (mRNA) was extracted out of all samples using a Nucleospin miRNA kit (Macherey-Nagel GmbH & Co. KG, Duren, Germany) according to the manufacturer’s instructions. Samples were disrupted with mortar and pestle in liquid nitrogen, and homogenized in lysis buffer by passing the lysate through a 23-gauge (0.64 mm) needle five times. Quantity of RNA was estimated with a NanoDrop ND-1000 spectrophotometer (NanoDrop Technologies Inc., Wilmington), and quality was further evaluated by agarose (1%) gel electrophoresis and an Agilent 2100 Bioanalyzer using the Agilent RNA 6000 Nano Kit. All samples had an RNA Integrity Number (RIN) value between 8.9 and 9.9.

### RNA sequencing (RNA-seq)

The transcriptome was assessed by sequencing a pool of mRNA of all developmental stages at the Norwegian Sequencing Centre, Oslo, Norway, using Illumina HiSeq *vs.* 2500. Therefore, paired-end libraries were prepared from a pool of eight developmental stages in total, and paired-end sequenced over one Illumina HiSeq lane. For differential expression analysis, paired-end libraries of four different developmental stages and three biological replicates of each stage, *i.e.*, middle gastrula (22:50 hpf), gastrula complete/onset of neurulation (30.30 hpf), mass hatching (102:15 hpf), and 2 dph (154:20 hpf) were prepared and sequenced on the Illumina MiSeq instrument using the TrueSeq *vs.* 3-600 kit (Supplemental Material, Figure S1). The use of multiplex identifier (MID) tags allowed the distinction among RNA of different stage and replicates.

### Quality control and assembly of reference transcriptome

Quality control was assessed using open source software FastQC (version 0.10.0; http://www.bioinformatics.babraham.ac.uk/projects/fastqc) and low quality reads were removed applying Trimmomatic software (Bolger *et al.* 2014). Sequences of all mixed eight stages obtained by Illumina HiSeq sequencing, as well as of the four stages obtained by Illumina MiSeq sequencing for differential expression analysis, were assembled into a reference transcriptome using Trinity version 2012-06-08 (Grabherr *et al.* 2011). Low abundance transcripts were excluded from the reference transcriptome in order to avoid false assembly products by applying RSEM [version 1.2.3; (Li and Dewey 2011)] filters FPKM and TPM.

### Differential expression analysis

Differential expression analysis comprised four developmental stages ranging from early to late stages of prelarval development and was carried out by paired-end sequencing of the constructed Illumina cDNA libraries on an Illumina MiSeq platform. The reads were mapped to the generated reference transcriptome using Bowtie (Langmead *et al.* 2009; Langmead and Salzberg 2012), with its default setting for maximum mismatches for each read. For quantification of read abundance, RSEM [version 1.2.3; (Li and Dewey 2011)] was applied. Transcripts representing less than once per million mappable reads were excluded from further downstream analysis. Evaluation of obtained data and differential expression between different stages was assessed using R Bioconductor package DESeq2 (Anders and Huber 2010). A transcript was considered as significantly differentially expressed with log_2_ fold change > |2| and padj < 0.005 in at least one stage compared with other stages. Significantly differentially expressed transcripts were further partitioned according to their expression pattern using a principal component analysis (PCA) clustering method in R statistical package (version 3.0.2).

### Annotation and classification

Filtered sequences were submitted to the nonredundant (nr) nucleotide database (Blastn) as well as to the nr protein database (Blastx) of the NCBI GenBank database. Further gene ontology (GO) analysis of positive blast matches was performed applying the free available Blast2GO software (Conesa and Götz 2008), where sequences are first mapped to GO annotations and subsequently annotated with their respective GO term. Successfully annotated transcripts were used for the generation of the taxonomic distribution of the top Blast matches and enrichment analysis. The latter was performed applying the Fischer’s Exact Test tool in Blast2GO with term filter modes “FDR” and “pval”; term filter values: FDR < 0.05 and/or *P* < 0.05, as well as two-tailed test. Transcripts upregulated during the first two stages, transcripts upregulated in the last two stages, as well as transcripts of two modules obtained by network analysis (see below), were used as test data sets that were compared to the annotated transcriptome (reference data set).

### Coexpression network construction

A coexpression network was generated according to the instructions of the WGCNA package in R (Langfelder and Horvath 2008). In brief, samples were clustered in order to detect outliers and, subsequently, a power of 12 and module size of 15 was chosen to generate scale-free topology networks. Network visualization of highly connected hub transcripts, defined in the present study of an edge weight value > 0.4, was performed applying the network visualization software Cytoscape.

### Data availability

Raw data have been deposited in the NCBI Short Read Archive (SRA) database (http://www.ncbi.nlm.nih.gov/sra/) under the accession number SRP069853. Assembled data were submitted to the Transcriptome Shotgun Assembly (TSA) database of NCBI under the accession number TSA SUB1335468. Assembled data have been deposited at DDBJ/EMBL/GenBank TSA under the accession GEUL00000000. The version described in this paper is the first version, GEUL01000000. 

## Results

### Transcriptome sequencing of Atlantic sturgeon developmental stages

**Figure 1:**
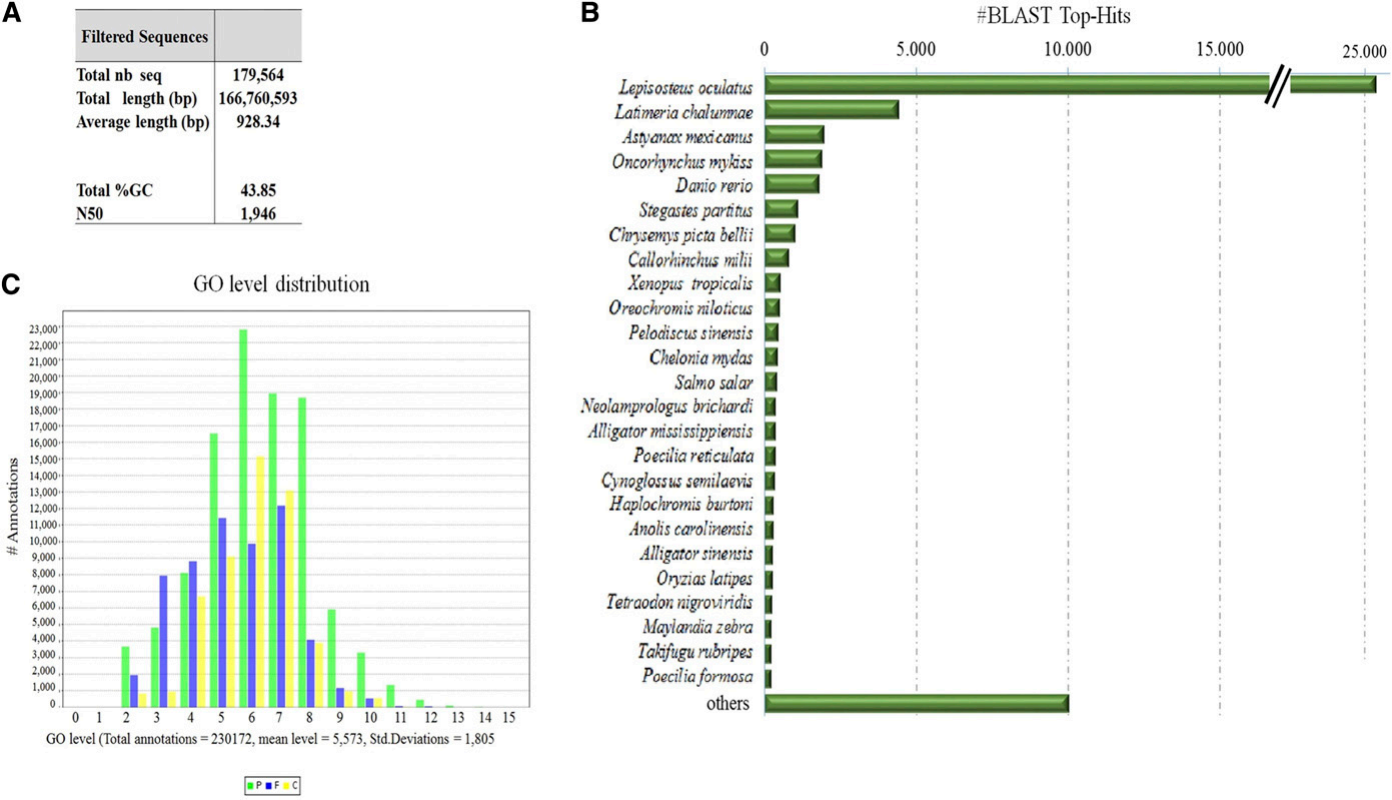
Summary of Blast2GO analysis. (A) Overview of obtained transcript number. (B) Histogram of the taxonomic distribution of the top Blast matches. (C) Distribution of gene ontology (GO) category.

To generate a robust reference transcriptome for the Atlantic sturgeon, a pool of eight developmental stages was submitted to Hiseq Illumina as well as to Illumina MiSeq sequencing, resulting in > 190 million raw reads. After trimming of low quality reads, ∼175 million reads (∼75%) remained for constructing the reference transcriptome assembly (Table 1). Assembly of all trimmed sequencing reads resulted in 441,209 transcripts with an average length of ∼711 bp and N50 of 1274 bp. Stringent RSEM filtering was applied, resulting in a total of 179,564 transcripts (Figure 1A and Figure S2). Robust reference transcriptome avoiding false assembly products is of importance for subsequent gene annotation and detection of differentially expressed transcripts. Obtained Miseq reads accounted for 31% of the transcripts belonging to the generated reference transcriptome. As the generated MiSeq reads of the four developmental stages for differential expression study constituted only 9% of the total number of reads used to generate the reference transcriptome (Table 2), the percentage of successfully mapped reads is a noteworthy result, showing the power of MiSeq sequencing for expression analysis as long as a suitable reference transcriptome is available.

**Table 1:** Overview of obtained sequencing reads

Sample	Input Read Pairs	Both Surviving (%)	Forward Only (%)	Reverse Only (%)	Dropped (%)
Pool 1	83,164,317	79,106,275 (95.12)	3,875,370 (4.66)	146,708 (0.18)	35,964 (0.04)
Pool 2	83,104,219	78,988,021 (95.05)	3,935,076 (4.74)	143,961 (0.17)	37,161 (0.04)
Middle gastrula a	1,704,737	1,219,891 (71.56)	480,352 (28.18)	1182 (0.07)	3312 (0.19)
Middle gastrula b	1,759,975	1,271,777 (72.26)	483,315 (27.46)	1462 (0.08)	3421 (0.19)
Middle gastrula c	2,007,739	1,629,840 (81.18)	372,199 (18.54)	1865 (0.09)	3835 (0.19)
Onset of gastrulation a	1,806,347	1,352,213 (74.86)	449,530 (24.89)	1178 (0.07)	3426 (0.19)
Onset of gastrulation b	4,256,369	2,959,524 (69.53)	1,280,399 (30.08)	2995 (0.07)	13,451 (0.32)
Onset of gastrulation c	4,049,259	3,011,472 (74.37)	1,026,308 (25.35)	2796 (0.07)	8683 (0.21)
Hatching a	1,305,484	921,386 (70.58)	380,613 (29.15)	1117 (0.09)	2368 (0.18)
Hatching b	1,575,899	1,103,488 (70.02)	468,456 (29.73)	1296 (0.08)	2659 (0.17)
Hatching c	1,228,471	830,425 (67.60)	395,148 (32.17)	865 (0.07)	2033 (0.17)
2 dph a	1,325,315	916,075 (69.12)	405,919 (30.63)	947 (0.07)	2374 (0.18)
2 dph b	1,230,047	825,250 (67.09)	401,289 (32.62)	1000 (0.08)	2508 (0.20)
2 dph c	1,524,312	1,066,236 (69.95)	454,420 (29.81)	1146 (0.08)	2510 (0.16)

dph, days posthatching.

**Table 2:** Overview of assembled and mapped sequencing reads

Sample	Number of Reads	% of Total Number of Reads	Number of Unique Transcripts Mapped onto Reference Transcriptome per Sample	% of the Reference Transcriptome Represented in the Unique Sample Reads
Pool 1	79,106,275	45.34	164,130	91.37
Pool 2	78,988,021	45.28	163,697	91.13
Middle gastrula a	1,219,891	0.70	12,736	7.09
Middle gastrula b	1,271,777	0.73	12,301	6.85
Middle gastrula c	1,629,840	0.93	4649	2.59
Onset of gastrulation a	1,352,213	0.78	6234	3.47
Onset of gastrulation b	2,959,524	1.70	26,883	14.97
Onset of gastrulation c	3,011,472	1.73	17,538	9.76
Hatching a	921,386	0.53	22,852	12.72
Hatching b	1103,488	0.63	23,566	13.12
Hatching c	830,425	0.48	23,279	12.96
2 dph a	916,075	0.53	18,372	10.23
2 dph b	82,525	0.05	19,769	11.01
2 dph c	1,066,236	0.61	22,501	12.53
Subtotal HiSeq	158,094,296	90.62	-	-
Subtotal MiSeq	16,364,852	9.38	55,214	30.74
Total	174,459,148	100	179,564	100

dph, days posthatching.

### Gene identification and annotation

Filtered transcripts were submitted to the NCBI GenBank database nr for blastn and blastx search, as well as to Blast2Go for gene annotation. Species tree distribution generated by Blast2Go showed that most top matches belonged to the spotted gar (*Lepisosteus oculatus*), followed by the coelacanth (*Latimeria chalumnae*) found in the West Indian Ocean (Figure 1B). Significant blastn matches were found for 58,408 transcripts (∼37%). GO terms were successfully assigned to 36,408 transcripts with the GO term “Biological Process” comprising more transcripts than the terms “Molecular Function” and “Cellular Component” (Figure 1C). For further downstream analysis such as enrichment analysis, the GO term “Biological Process” was assessed. GO distribution for each term is illustrated in Figure S3.

### Assessment of differential expression

**Figure 2:**
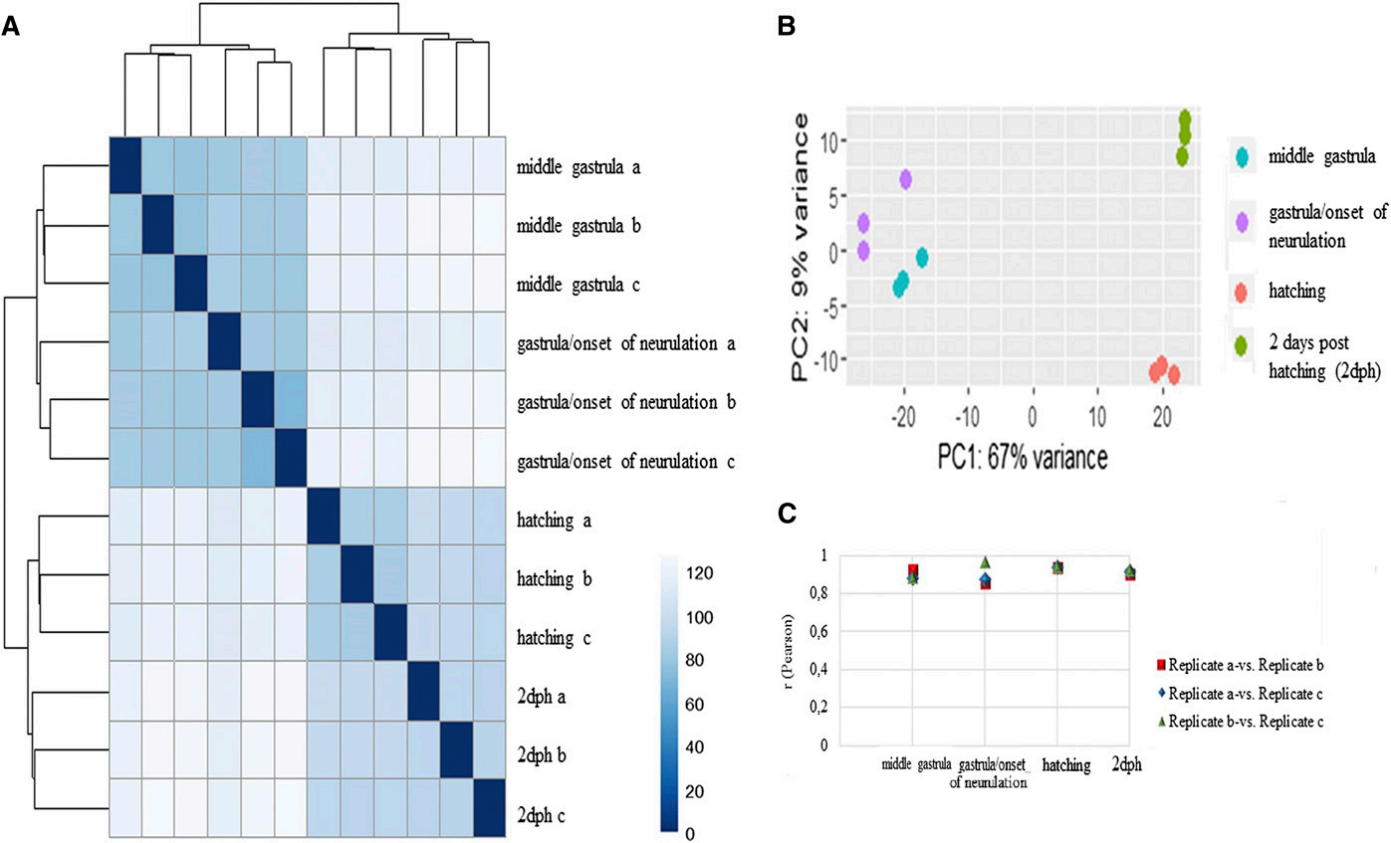
(A) Sample-to-sample distances. Heatmap generated with DeSeq2 software packages showing the Euclidean distances between the samples. (B) PCA analysis of significantly regulated transcripts in at least one stage. Different colors denote the investigated stages. PCA plot was generated with the *ggplot2* library. (C) Pearson’s correlation coefficient analysis of all significantly regulated transcripts in at least one stage. The Pearson *r* values of all possible combinations are shown at the *y* axes while the stages are given at the *x*-axes. dph, days posthatching; PCA, principal component analysis.

MiSeq reads obtained out of four developmental stages were mapped onto the generated reference transcriptome (Table 2). Transcripts considered as differentially expressed in any of the four stages accounted for 1627 transcripts. Pairwise comparison of the number of obtained differentially expressed transcripts with two different threshold values are shown in Table 3. In addition, the quality of the obtained data are shown in Figure 2, where in Figure 2A the sample-to-sample Euclidean distance is shown in form of a heatmap and in Figure 2B and Figure S4A samples were clustered by principal component analysis PCA. Finally, Pearson’s correlation coefficient analysis was performed and is illustrated as a graph in Figure 2C. All three methods show the clustering of biological replicates as well as a clear separation of the first two stages (middle gastrula and gastrula/onset of neurula) from the latter two (hatching and 2 dph). The same expression pattern is also seen after hierarchical clustering analysis of all detected significant transcripts illustrated in the form of a heatmap (Figure S4B).

**Table 3:** Number of differentially expressed transcripts at three different significant thresholds

DE	padj < 0.05	padj < 0.005	padj < 0.005 Log2 Fold Change > |2|
Middle gastrula *vs.* onset of gastrulation	87	41	27
Middle gastrula *vs.* hatching	1801	706	590
Middle gastrula *vs.* 2 dph	1977	745	653
Onset of gastrulation *vs.* hatching	216	1067	886
Onset of gastrulation *vs.* 2 dph	2694	1287	1097
Hatching *vs.* 2 dph	114	48	28

DE, differentially expressed transcripts; padj, adjusted *P*-value; dph, days posthatching.

### Coexpression network

**Figure 3:**
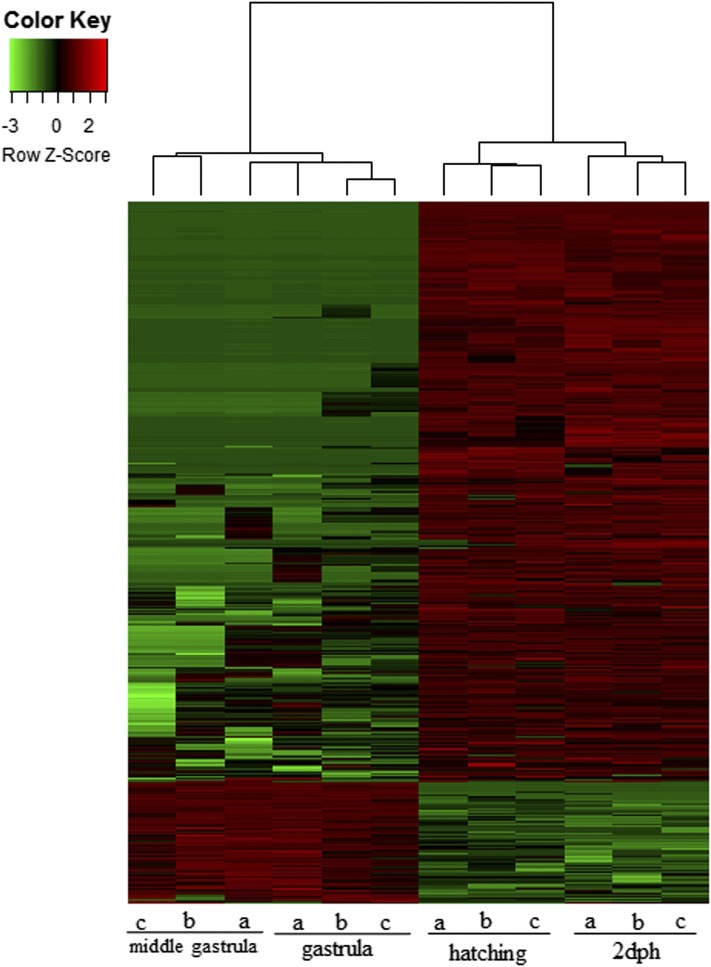
Heatmap of expression pattern and hierarchical clustering of differentially expressed transcripts belonging to module 1. Coexpression network was generated according to the instructions of the WGCNA package in R (Langfelder and Horvath 2008). Samples were clustered in order to detect outliers and subsequently a power of 12 and module size of 15. Expression patterns of the largest module (module 1), accounting for 1351 transcripts, is shown. dph, days posthatching.

**Figure 4:**
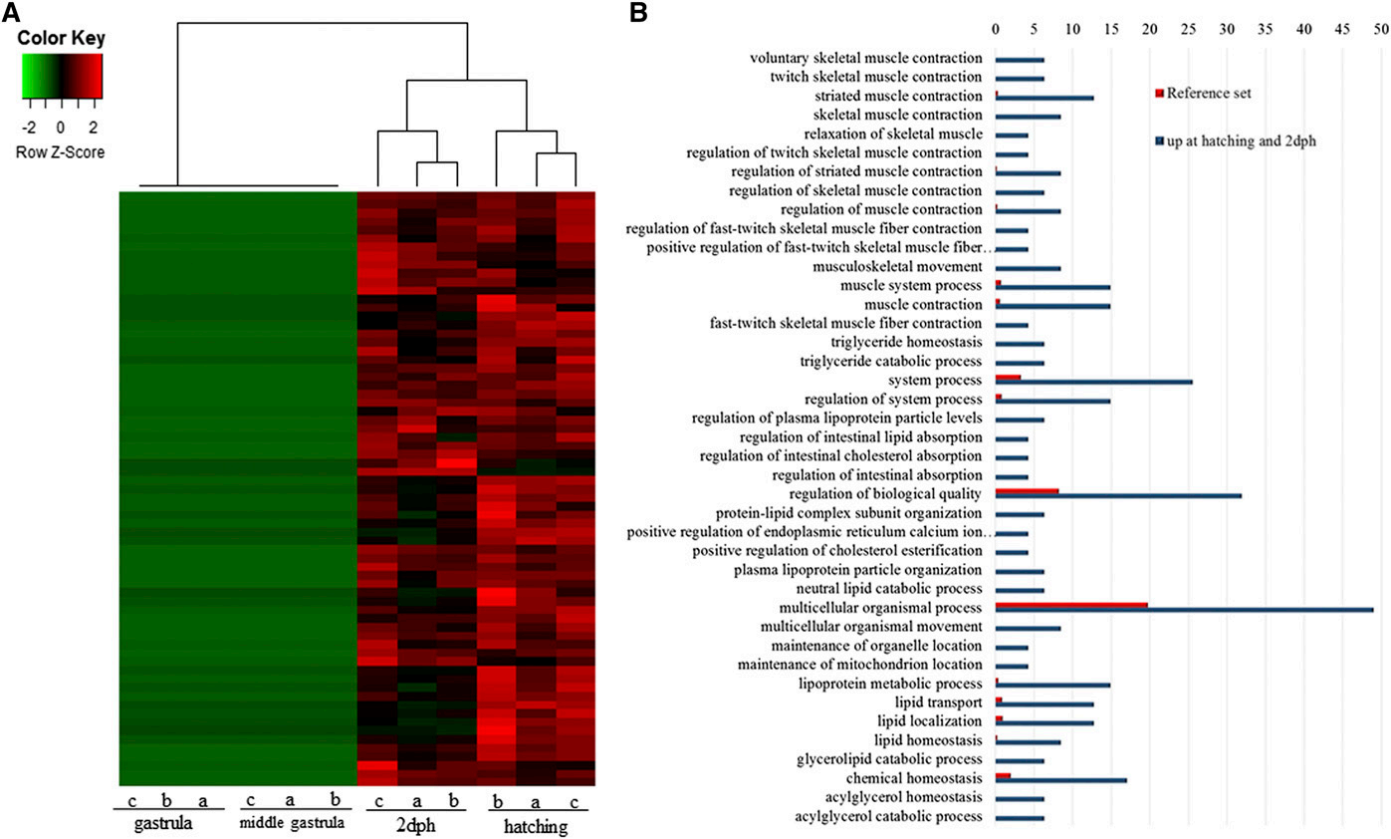
(A) Heatmap of expression pattern and hierarchical clustering of transcripts detected only at the hatching and 2 dph stages. Only transcripts expressed at all three biological replicates of the hatching and 2 dph stages, but with no expression in all three biological replicates at the middle gastrula and gastrula stages, are shown. (B) Histogram of the most significantly enriched GO terms belonging to the category “Biological Process” from the GO enrichment analysis of transcripts only present at the two later stages. The significance of each GO term was determined based on the *P*-value < 0.005 and FDR value < 0.005. The reference set is denoted in red, while the blue color denotes transcripts that were present only at the hatching and 2 dph stages. dph, days posthatching; FDR, false discovery rate; GO, gene ontology.

**Figure 5:**
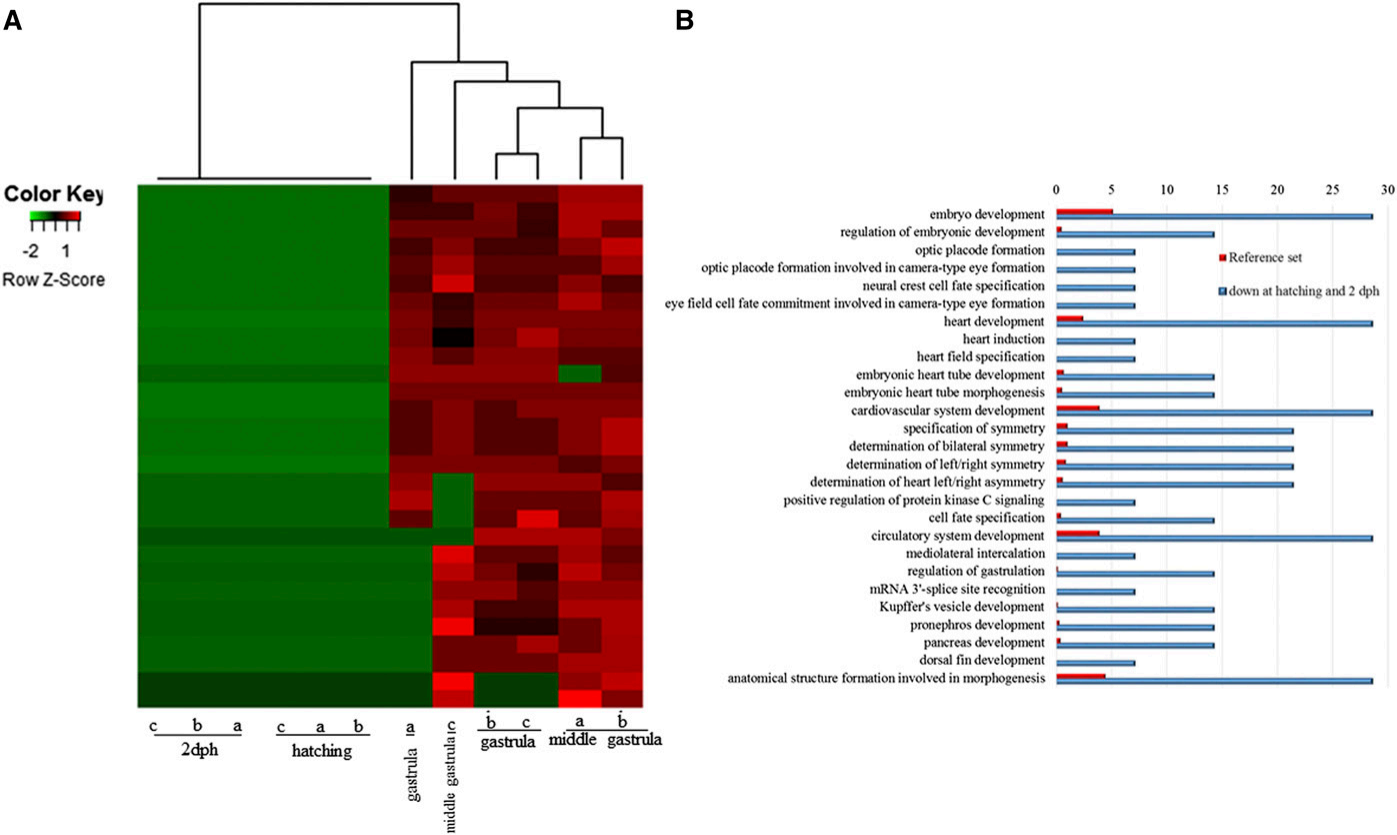
(A) Heatmap of expression pattern and hierarchical clustering of transcripts not detected at the hatching and 2 dph stages. Only transcripts with no expression in all three biological replicates of the hatching and 2 dph stages are shown. (B) Histogram of the most significantly enriched GO terms belonging to the category “Biological Process” from the GO enrichment analysis of transcripts that were not detected at the two later stages. The significance of each GO term was determined based on the p-value < 0.005. The reference set is denoted in red, while the blue color denotes transcripts that were downregulated at the hatching and 2 dph stages. dph, days posthatching; GO, gene ontology; mRNA, messenger RNA.

A coexpression network of significantly differentially expressed transcripts resulted in six modules with the largest one, module 1, comprising most of the submitted transcripts (1351). Their expression profiles show a clear separation of transcripts at the first two stages (“middle gastrula” and “gastrula/onset of neurulation”) from transcripts at the last two stages (“hatching” and “2 dph”) by either up- or downregulation (Figure 3). Transcripts exclusively upregulated at the hatching and 2 dph stages (Figure 4A) added up to a total of 69, and enrichment analysis resulted in GO terms comprising mainly muscle contraction as well as muscle movement (Figure 4B). On the other hand, enrichment analysis with any transcripts not expressed at all at the hatching and the 2 dph stages, regardless of significance (Figure 5A), resulted in GO terms related explicitly to embryo development (Figure 5B).

**Figure 6:**
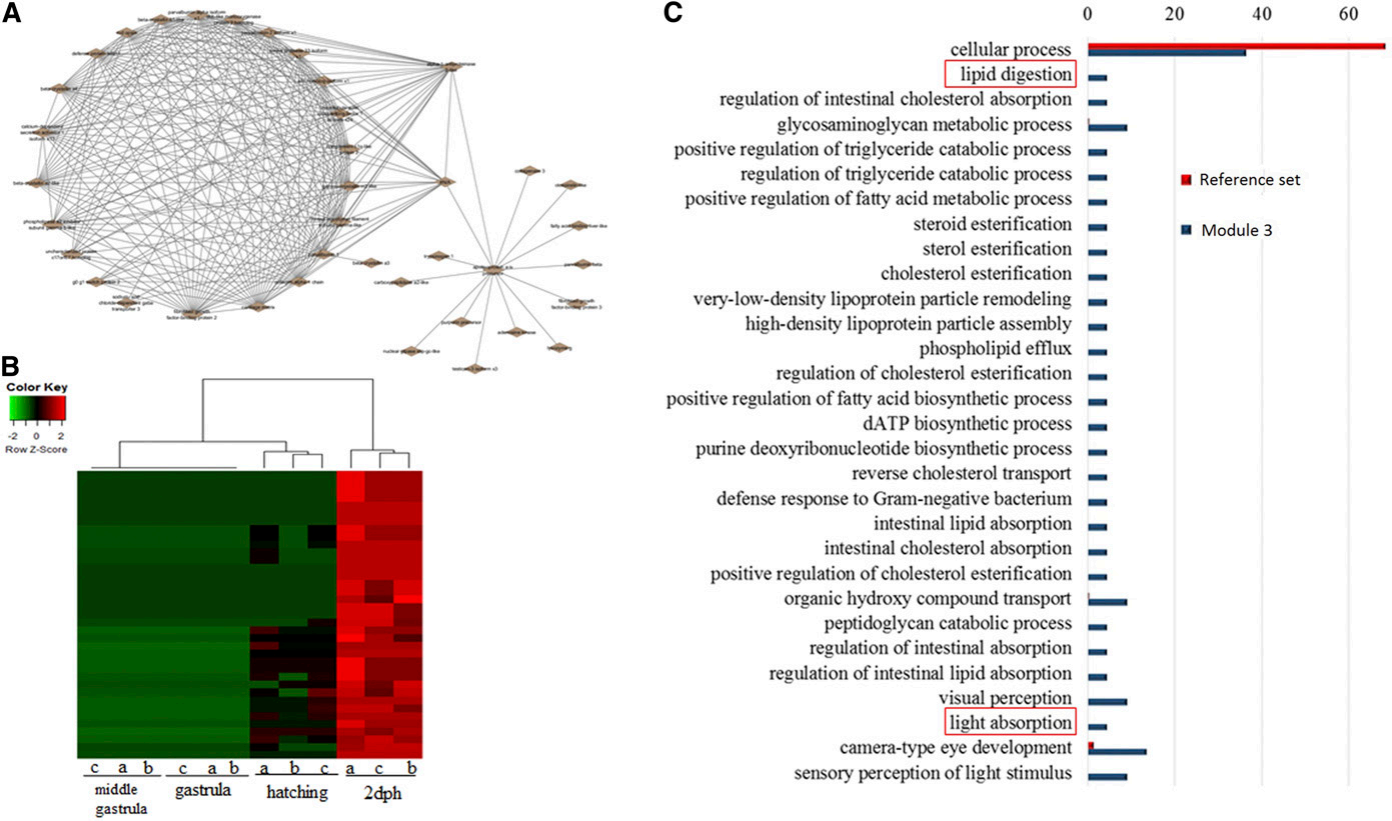
(A) Network of module 3. Coexpression network was generated according to the instructions of the WGCNA package in R (Langfelder and Horvath 2008). Samples were clustered in order to detect outliers and subsequently a power of 12 and module size of 15. The network of module 3 is displayed. (B) Heatmap of expression patterns and hierarchical clustering of transcripts in module 3. Expression patterns of module 3-accounting 34 transcripts are shown. (C) Histogram of the most significantly enriched GO terms belonging to the category “Biological Process” from the GO enrichment analysis of transcripts of the network shown in (A) and (B). The significance of each GO term was determined based on the *P*-value < 0.005. The reference set is denoted in red, while the blue color denotes transcripts that are presented in the network of module 3. dATP, deoxyadenosine triphosphate; dph, days posthatching; GO, gene ontology.

**Figure 7:**
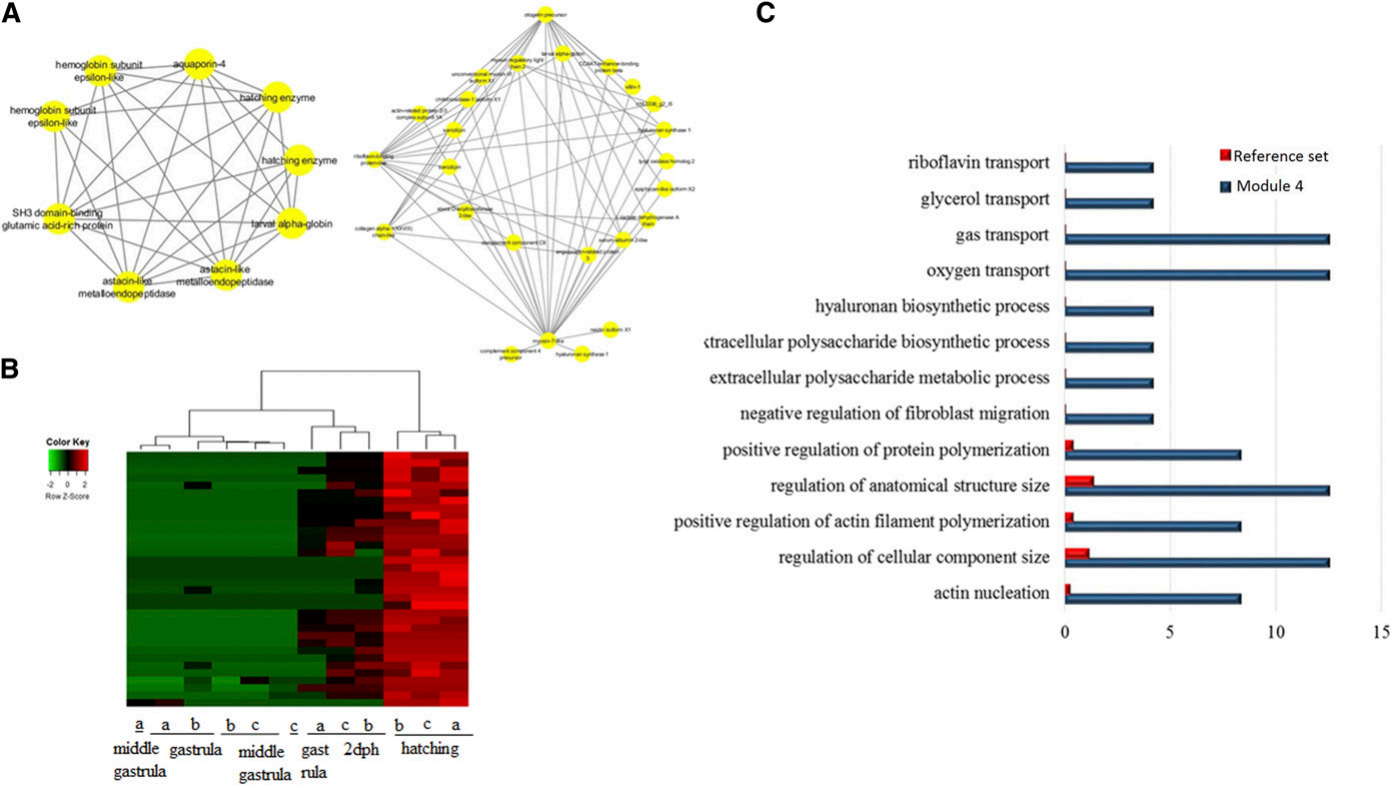
(A) Network of module 4. Coexpression network was generated according to the instructions of the WGCNA package in R (Langfelder and Horvath 2008). Samples were clustered in order to detect outliers and subsequently a power of 12 and module size of 15. The network of module 4 is displayed. (B) Heatmap of expression patterns and hierarchical clustering of transcripts in module 4. Expression patterns of the module 4-accounting 37 transcripts are shown. (C) Histogram of the most significantly enriched GO terms belonging to the category “Biological Process” from the GO enrichment analysis of transcripts of the network shown in (A) and (B). The significance of each GO term was determined based on the *P*-value < 0.005. The reference set is denoted in red, while the blue color denotes transcripts that are presented in the network of module 4. dph, days posthatching; GO, gene ontology.

The remaining transcripts formed four smaller modules: module 2 comprising 80 transcripts, module 3 with 70, module 4 with 48 transcripts, and modules 5 and 6 with 32 and 12 transcripts respectively. Modules 2, 5, and 6 did not show distinct expression patterns and were thus not further examined. In the present work, modules 3 and 4 are illustrated using cytoscape software. Further cytoscape filtering using a node threshold value of > 0.4 and > 0.45 was applied, respectively. After cytoscape filtering, module 3 comprised 38 nodes with three main hubs (Figure 6A). Illustrated transcripts in Figure 6A showed increased transcription at the last stage (2 dph) (Figure 6B). Enrichment analysis revealed GO terms linked to lipid metabolism as well as in GO terms related to the development of visual characteristics (Figure 6C). Special attention was paid to module 4 with 34 transcripts after filtering (Figure 7A). For this module, two separate networks were obtained with the smaller one comprising transcripts known to be involved in the hatching process, but also transcripts not yet described as having an active role during hatching such as aquaporin. Expression is illustrated as a heatmap in Figure 7B, revealing an increased transcription level at the hatching stage. Enrichment analysis revealed GO terms linked to molecule transport such as riboflavin transport, as well as regulatory processes (Figure 7C).

## Discussion

Molecular data for sturgeons are still scarce in spite of the rapid advances in sequencing technologies. To our best knowledge, up until today, RNA-seq has been performed in gonadal tissue of the Chinese sturgeon (*A. sinensis*) (Yue *et al.* 2015), in the gonads and brain of the Adriatic sturgeon (*A. naccarii*) (Vidotto *et al.* 2013), in the lake sturgeon (*A. fulvescens*) (Hale *et al.* 2009), as well as in the Amur sturgeon (*A. schrenckii*) focusing on microRNA (Yuan *et al.* 2014). Regarding developmental stages, only one other recent study has investigated the transcriptome of five different developmental stages in the Siberian sturgeon (*A. baerii*) and generated an assembled transcriptome with 278,167 transcripts (Song *et al.* 2015). In the present study, the reference transcriptome of the Atlantic sturgeon (*A. oxyrinchus*) was generated combining RNA-seq data obtained out of a pool of eight different developmental stages and RNA-seq data of four developmental stages in total (Table 2). After stringent filtering, a total of 179,564 unique back-mapped transcripts were obtained. This number is comparable to the work in *A. baerii*, as here the amount of initial raw reads were higher compared to the present work (38 and 28 Gb, respectively). In addition, in the present study, more stringent filters were applied. Further characterization of the reference transcriptome by Blast2GO analysis is summarized in Figure 1 with the blast “top hit species distribution” (Figure 1B) having as first match the recently sequenced spotted gar (*Le. oculatus*) (Braasch *et al.* 2016), followed by the African coelacanth (*La. chalumnae*), and the Mexican blind cave fish (*Astyanax mexicanus*). This result was expected taking into account the taxonomic position of sturgeons and validates the obtained reads. Concerning GO terms, the most successful GO annotation was obtained for the GO category “Biological process” (Figure 1C), with the majority of the transcripts mapping to “cellular process,” “single-organism process,” and “metabolic process” (Figure S3).

In contrast to a previous study in *A. baerii*, a clear separation of earlier to later developmental stages was observed (Figure 2, A and B and Figure S4, A and B). Similar to the present work, gene expression studies regarding embryogenesis of *Fundulus heteroclitus* also highlighted significant differences in gene expression between pre- and posthatching (Bozinovic *et al.* 2011). A possible explanation for the discrepancy with the study of *A. baerii* could be the use of more subsequent developmental stages in *A. baerii*, while in the present work earlier and also denser developmental stages were studied. Further network analysis to investigate expression patterns underlined the clear separation of those two groups by one large module, where most of the transcripts were shown to be more highly expressed in the two later stages (Figure 3). Austere filtering by looking only at transcripts exclusively expressed at stages 10 (hatching) and 16 (2 dph), with > 10 transcripts mapped, amounted to 69 and are illustrated in the form of a heatmap in Figure 4A. Enrichment analysis revealed that transcripts expressed at the latter two stages are mainly involved in the proper function of muscles (*e.g.*, skeletal muscle proteins) (Figure 4B), whereas transcripts found not to be expressed at all in the last two stages under study (Figure 5A) are enriched in GO terms linked to developmental processes (*e.g.*, embryo development) (Figure 5B). However, respective transcripts having increased expression at the first two stages are not as congruent in all three replicates as was shown for those transcripts being exclusively upregulated at the hatching and 2 dph stages. Nevertheless, enrichment analysis resulted in GO terms linked to embryo development, as well as symmetry determination and organ development (Figure 5B). These results also show that stringent filtering may compensate one biological replicate that is giving weak accordance with the other two. Expression patterns and the outcome of the enrichment analysis reveal that developmental processes are completed up until hatching, while thereafter it is mainly genes encoding structural proteins that are of importance. This has also been shown in the zebrafish (*Danio rerio*) (Mathavan *et al.* 2005), but also in other nonmodel teleost species like the Atlantic halibut (Bai *et al.* 2007), the Atlantic bonito (*Sarda sarda*) (Sarropoulou *et al.* 2014), and the gilthead sea bream (*Sparus aurata*) (Sarropoulou *et al.* 2005). The other two modules of interest, obtained by coexpression network analysis, are modules 3 and 4. Module 3 (Figure 6A) comprises transcripts highly expressed at 2 dph (Figure 6B). After hatching, larvae start feeding on planktonic and benthic organisms and, after yolk sack absorption, actively feed only on benthic organisms. In addition, soon after hatching, eye pigmentation starts in order to perceive light. The histogram of the most enriched GO terms comprises both aspects important to the larval process: lipid digestion as well as light absorption (Figure 6C). Similar results have been obtained in the gilthead sea bream (*S. aurata*), where genes involved in eye pigmentation were upregulated at the stage of mouth opening (Sarropoulou *et al.* 2005). Module 4 (Figure 7A) revealed transcripts expressed only at the hatching stage, which also formed a small subgroup of transcripts after hierarchical cluster analysis (Figure S4). Key transcripts (highest expression as well as highest edge values in the network analysis) were determined as hatching enzymes, aquaporin, as well as globins (Figure 7A). Further enrichment analysis resulted in 13 enriched GO terms comprising the GO term “riboflavin transport.” In fowl flocks, as far back as the late 1970s, it was shown that riboflavin is required for proper egg production and hatchability (Anon 1979). The GO terms glycerol transport as well as regulation of cellular component size pinpoint to the swelling of the egg during hatching and comprise the transcript encoding aquaporin. Teleost embryos are protected from environmental parameters by an egg envelope. At hatching, this envelope is partially digested. Studies investigating the mechanism of egg envelope digestion have proposed different mechanisms according to the phylogenetic position of each species. It has been suggested that eggs of basal teleosts like the Japanese eel (*Anguillarum anguillarum*) first swell and soften their egg envelope via proteolytic enzymes in order to subsequently be torn by movements of the embryo (Sano *et al.* 2011). In the present study, besides the identification of hatching enzymes upregulated exclusively at the hatching stage, we also identified a transcript encoding aquaporin-4 that is upregulated only at hatching (Figure 7B). Previous studies have shown that aquaporins are involved in osmoregulation (Finn and Cerdá 2011) as well as in egg hydration of marine fish to control egg survival and dispersion in the ocean (Fabra *et al.* 2005). The finding of strong upregulation of an aquaporin transcript at the hatching stage, and the results of previous studies revealing an osmoregulative function, suggests that aquaporin-4 plays an important role during hatching as eggs of basal teleost, as previously mentioned, first swell prior to hatching (Sano *et al.* 2011).

### Conclusions

The manuscript reports the characterization of the Atlantic sturgeon (*A. oxyrinchus*) transcriptome during early development up until 2 dph. It shows differential expression among four distinguished developmental stages with stage-specific expression patterns. We further show that transcripts encoding genes involved in visual perception as well as in lipid digestion are significantly enriched at the 2 dph stage, while transcripts expressed at the last two stages encode genes important for muscle contraction. The present manuscript further produces evidence for the putative involvement of aquaporin genes along with the already described hatching enzymes during the process of hatching. Overall, the obtained data set along with the transcript characterization and differential expression results will significantly contribute to sturgeon conservation and aquaculture.

## Supplementary Material

Supplemental material is available online at www.g3journal.org/lookup/suppl/doi:10.1534/g3.116.036699/-/DC1.
