# Supplementary figures and images for: Expression Patterns of Atlantic Sturgeon (Acipenser oxyrinchus) During Embryonic Development
Source: G3 (Bethesda). 2016 Dec 14;7(2):533–42. doi: 10.1534/g3.116.036699 (PMC5295599; doi:10.1534/g3.116.036699)

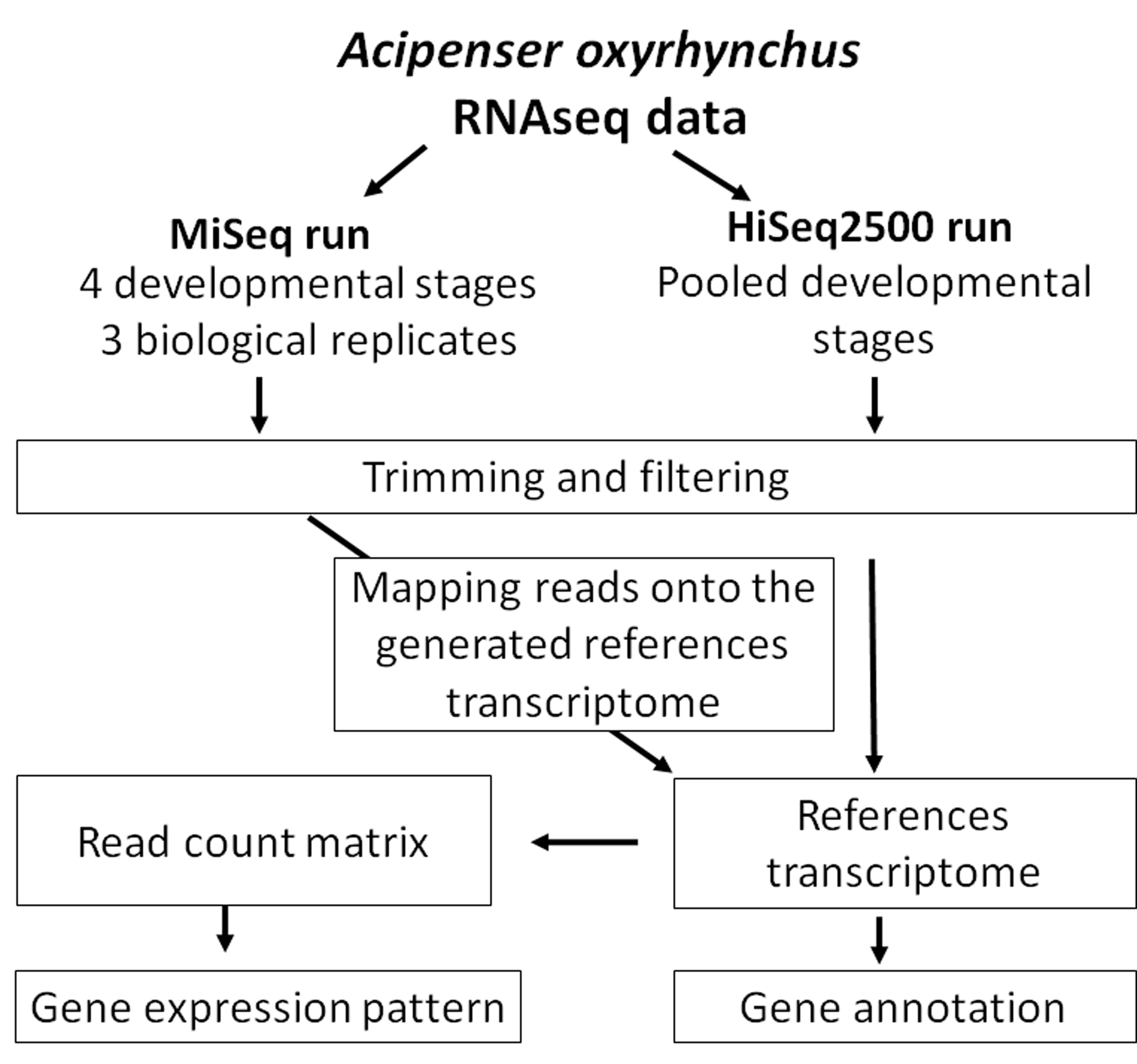

Supplement: Supplementary file 1 [file 533FigureS1.jpg]

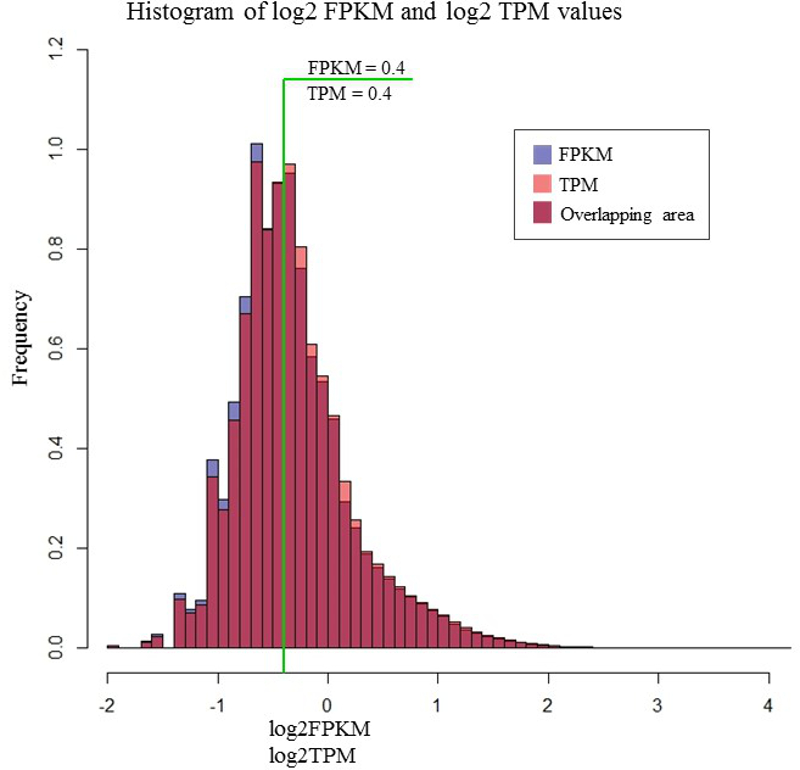

Supplement: Supplementary file 2 [file 533FigureS2.jpg]

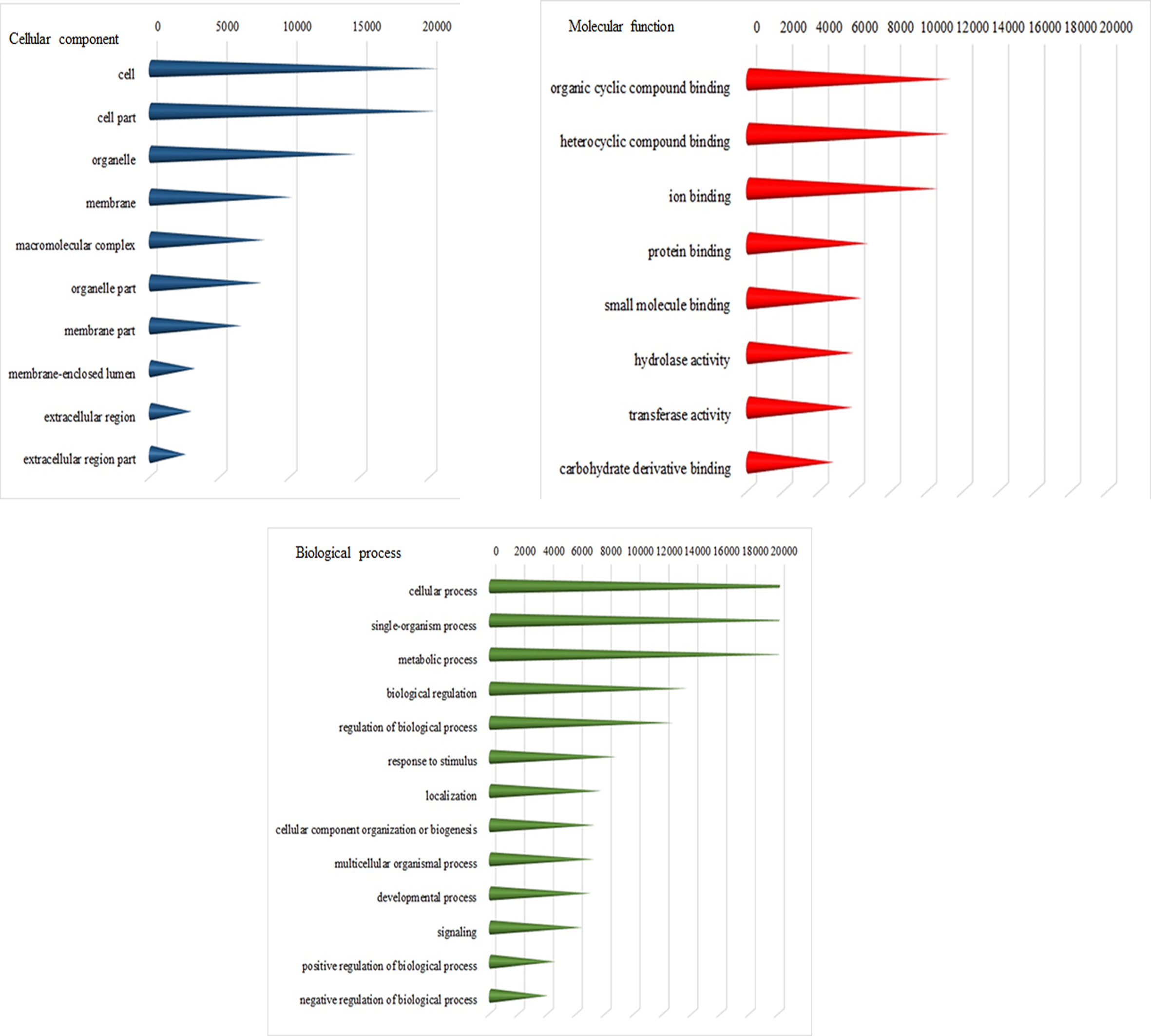

Supplement: Supplementary file 3 [file 533FigureS3.jpg]

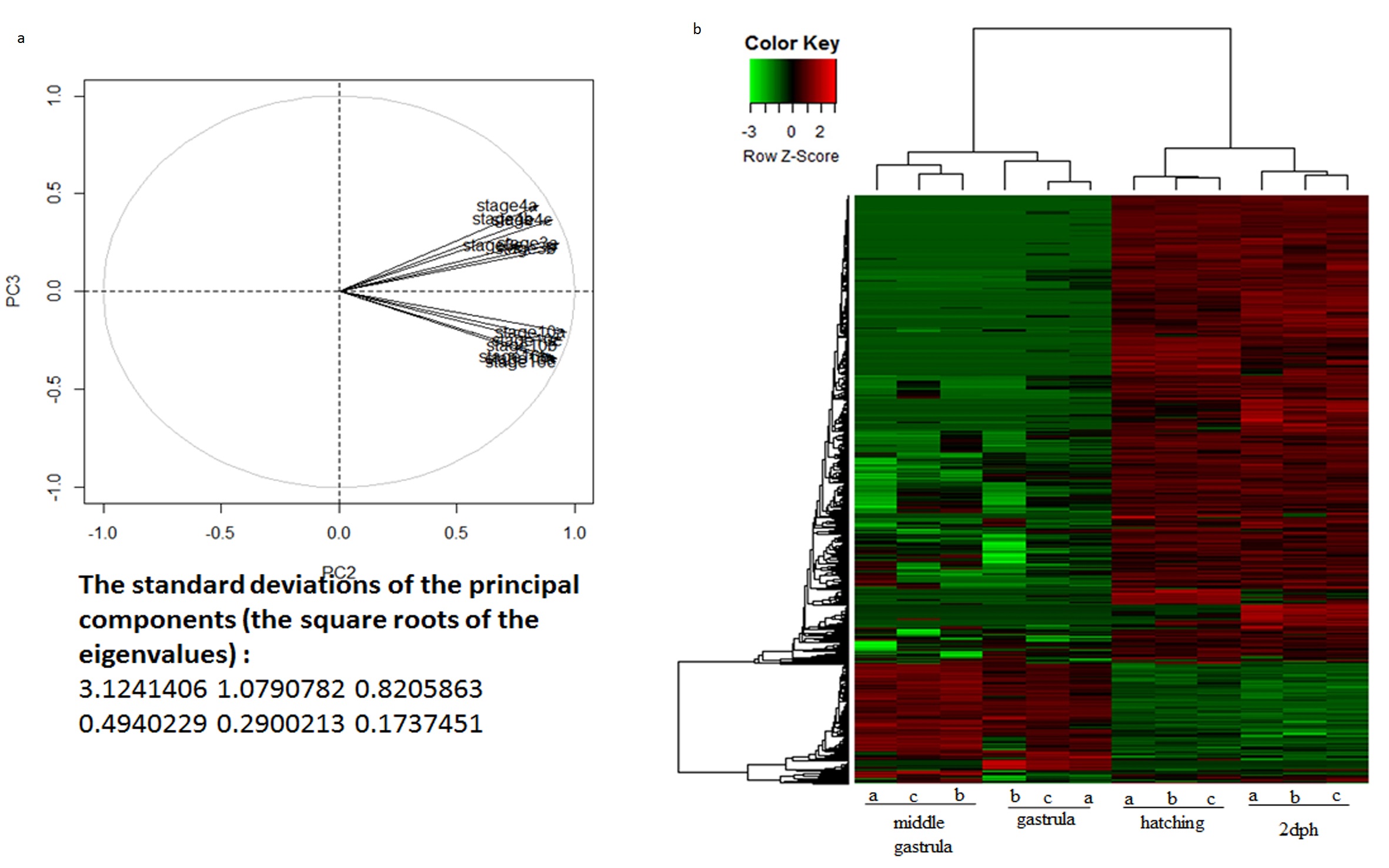

Supplement: Supplementary file 4 [file 533FigureS4.jpg]
